# Retinal Hemorrhage after SARS-CoV-2 Vaccination

**DOI:** 10.3390/jcm10235705

**Published:** 2021-12-05

**Authors:** Hyo Song Park, Yeojue Byun, Suk Ho Byeon, Sung Soo Kim, Yong Joon Kim, Christopher Seungkyu Lee

**Affiliations:** 1 Department of Ophthalmology, The Institute of Vision Research, Severance Hospital, Yonsei University College of Medicine, Seoul 03772, Korea; hyosong@yuhs.ac (H.S.P.); SHBYEON@yuhs.ac (S.H.B.); SEMEKIM@yuhs.ac (S.S.K.); 2 Kong Eye Clinic, Seoul 03157, Korea; yeojue@naver.com

**Keywords:** retinal vein occlusion, submacular hemorrhage, SARS-CoV-2, vaccine, ChAdOx1 nCoV-19, BNT162b2

## Abstract

To report retinal vein occlusion (RVO) and age-related macular degeneration (AMD)-related submacular hemorrhage developing after administration of SARS-CoV-2 vaccines, a single-center, retrospective observational case series was conducted. Clinical data including fundus photographs and optical coherence tomography (OCT) scans were reviewed. Twenty-three eyes of 21 patients were included with the median age at symptom presentation being 77 years (range: 51–85 years). Twelve eyes (52.2%) had submacular hemorrhage and 11 (47.8%) had RVO. Twelve patients (60.9%) had been vaccinated with the Pfizer vaccine (BNT162b2) and 8 with the AstraZeneca (ChAdOx1) vaccine. Sixteen patients (76.2%) experienced ocular disease exacerbation after the first vaccination and 4 (19.0%) after the second vaccination. The median visual acuity (logarithm of the minimal angle of resolution; logMAR) before symptom development was 0.76 (interquartile range: 0.27–1.23); the median logMAR at symptom presentation was 1.40 (interquartile range 0.52–1.70). The median time between vaccination and symptom exacerbation was 2.0 days (interquartile range: 1.0–3.0 days). Five patients (23.8%) underwent tests for hematological abnormalities, including the presence of anti-PF4 antibodies; all were negative. Further studies with larger patient group for evaluation of effect of SARS-CoV-2 vaccination on retinal hemorrhage are necessary.

## 1. Introduction

The ongoing coronavirus SARS-CoV-2 pandemic has caused 4 million deaths worldwide among 199 million cases [1]. Recently developed vaccines have been promptly administered on a scale never seen before. Some vaccines are DNA or mRNA vaccines [2,3], as opposed to conventional live-attenuated or viral-vectored vaccines, but were nevertheless approved by the Food and Drug Administration (FDA) and the European Medicines Agency (EMA) exceptionally quickly, given the grave global situation. Large-scale vaccination is ongoing worldwide.

In South Korea, administration of the SARS-CoV-2 adenoviral vector vaccines ChAdOx1 nCoV-19 (AstraZeneca) and Ad26.COV2.S (Johnson & Johnson), and the mRNA vaccines BNT162b2 (Pfizer-BioNTech) and mRNA-1273 (Moderna), commenced in February 2021. As of 31 July 2021 [4], over 17 million of the population of 52 million have received at least one injection, and almost 7 million have had finished their vaccination as indicated [4]. Most people were vaccinated with ChAdOx1 nCoV-19 or BNT162b2 (>10 and 4.9 million, respectively) [4]. Ad26.COV2.S was given to more than 1.1 million people and mRNA-1273 to 60,000 [4]. All of the vaccines are at least partially effective against SARS-CoV-2 [2,3,5,6,7]. However, few reports on vaccine safety have appeared, although some serious vaccine-related side-effects, including immune thrombotic thrombocytopenia (VITT), cerebral venous sinus thrombosis (CVST), and cardiomyopathy (all of which are life-threatening) were reported after mass inoculation began [8,9,10,11,12]. Although the mechanisms by which these conditions develop remain unclear, VITT patients seem to have antibodies against platelet factor 4 (anti-PF4 Ab); these mimic the effect of heparin by binding to a similar site on PF4, which allows PF4 to cluster into immune complexes that trigger Fcγ receptor IIa-dependent platelet activation [13]. The mechanism of cardiomyopathy may involve molecular mimicry of the spike protein of severe acute respiratory syndrome coronavirus-2 (SARS-CoV-2) and self-antigens, the triggering of pre-existing dysregulated immune pathways in certain individuals, an immune response to mRNA followed by activation of immunological pathways, and/or dysregulated cytokine expression [12].

With the advent of the B.1.617.2 (delta) variant, it has been suggested that further boosters may be required. This increases the need for ophthalmological studies on vaccine-related side-effects, where SARS-CoV-2 vaccination may become annual. Known intraocular side-effects include acute macular neuroretinopathy, central serous chorioretinopathy, reactivation of Vogt-Koyanagi-Harada disease, panuveitis, Descemet’s membrane endothelial keratoplasty (DMEK) graft rejection, retinal hemorrhage and vascular events including retinal arterial occlusion and retinal vein occlusion (RVO) [14,15,16,17,18,19,20,21,22]. Here, we present a unique case series of RVO, and age-related macular degeneration (AMD) exhibiting submacular hemorrhage, temporally related to administration of SARS-CoV-2 vaccines.

## 2. Materials and Methods

This single-center, retrospective observational case series adhered to the tenets of the Declaration of Helsinki and received approval from the institutional review board of Severance Hospital (IRB no. 4-2021-0737). Given the retrospective nature of the work, the need for informed patient consent was waived. We included new or exacerbated RVO in general and/or AMD-related submacular hemorrhage patients who developed symptoms after SARS-CoV-2 vaccination; the patients were evaluated between April and July 2021 at Severance Hospital. We retrieved data on age, gender, symptoms, laterality, diagnosis, and visual acuity from medical records. Medical and ocular histories prior to symptom development or exacerbation were reviewed. The date, type, and number of vaccinations were reviewed, and the interval between vaccination and symptom development was recorded. Where possible, an anti-PF4 antibody assay and blood tests including complete blood count (CBC) were performed, and the prothrombin time (PT) and activated partial thromboplastin time (aPTT) were recorded to determine whether there was a hematological abnormality. Treatments were also recorded as appropriate.

New or exacerbated RVO after vaccination was defined as a new vitreous or retinal hemorrhage developing with subjective symptom of decreased visual acuity within 28 days of vaccination that was clinically confirmed by at least three retinal specialists. New or exacerbated submacular hemorrhage after vaccination was defined as an unforeseen increase in the extent of hemorrhage with subjective symptom of decreased visual acuity within 28 days of vaccination that was clinically confirmed by at least three retinal specialists. All eyes underwent color fundus photography or ultra-wide-field fundus photography using a fundus camera (Optos PLC, Dunfermline, UK) and spectral-domain optical coherence tomography (OCT; Spectralis OCT; Heidelberg Engineering, Heidelberg, Germany). Where possible, OCT or fluorescein angiography was performed using a Spectralis HRA+OCT device (Heidelberg Engineering, Heidelberg, Germany). The images were evaluated in terms of the extent of vitreous or retinal hemorrhage. The means, medians, and standard deviations of the clinical features are presented. As the sample size was small, nonparametric tests were used to evaluate statistical significance; the data are presented as medians with interquartile ranges. 

## 3. Results

Twenty-three eyes of 21 patients (11 men and 10 women) were included. Patient demographics are demonstrated in Table 1. The median age at symptom presentation was 77 years (range: 51–85 years). All of the patients visited our clinic complaining of decreased visual acuity after SARS-CoV-2 vaccination. Most patients exhibited unilateral submacular hemorrhage or RVO, but two had bilateral disease (one patient had AMD in both eyes, and the other had AMD in the right eye and RVO in the left eye). Twelve eyes (52%) had AMD and eleven (48%) had RVO. Nine patients (43%) had diabetes mellitus and thirteen (62%) had hypertension. Twelve patients (61%) had been vaccinated with the Pfizer vaccine (BNT162b2) and eight with the AstraZeneca (ChAdOx1) vaccine. Seventeen patients (81%) experienced exacerbation of ocular disease after their first vaccination, and four (19%) after their second vaccination. Five patients (24%) were tested for hematological abnormalities including the presence of anti-PF4 antibodies. The median visual acuity (logarithm of the minimal angle of resolution; logMAR) before symptom development in 16 eyes was 0.76 (interquartile range: 0.27–1.23) and the value at symptom presentation was 1.40 (interquartile range 0.52–1.70). The median time between vaccination and symptom exacerbation was 2.0 days (interquartile range: 1.0–3.0 days).

### 3.1. Submacular Hemorrhage after SARS-CoV-2 Vaccination

**Figure 1:**
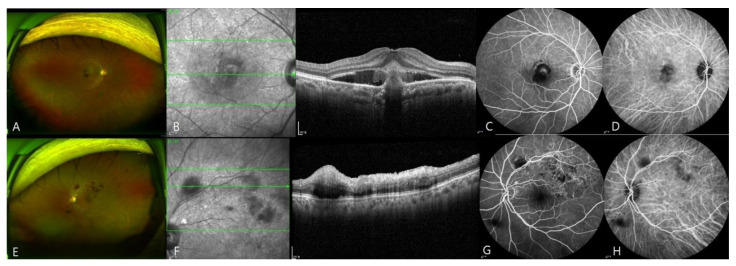
Images of a patient with submacular hemorrhage and retinal vein occlusion (RVO) in both eyes (eyes 7 and 19). The patient developed submacular hemorrhage in the right eye and RVO in the left eye at 2 weeks after the first injection of BNT162b2. (**A**–**D**), Wide-field fundus photograph, OCT scan, fluorescein angiograph, and ICG angiograph of the right eye. OCT revealed subretinal hemorrhage. (**E**–**H**), Ultra-wide-field fundus photograph, OCT scan, fluorescein angiograph, and ICG angiograph of the left eye. Fluorescence angiography revealed blocked fluorescence at the site of retinal hemorrhage.

**Figure 2:**
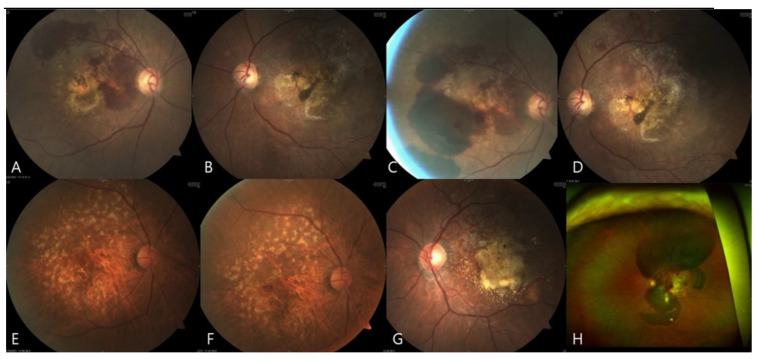
Images of representative eyes exhibiting submacular hemorrhage after vaccination. (**A**–**D**), Fundus photographs of eyes 1 and 2 before and after symptom exacerbation. This AMD patient developed subretinal hemorrhage in both eyes (**A**,**B**). Three days after the first dose of BNT162b2 and 1 week after photographs A and B were taken, the patient developed blurred vision, and fundus photographs taken on the following day revealed that the hemorrhage in both eyes had worsened (**C**,**D**). (**E**,**F**), Fundus photographs of eye 10 taken 4 weeks before and after symptom exacerbation. Two days after ChADOx1 vaccination, the patient experienced visual disturbance, and fundus photography revealed a new submacular hemorrhage. (**G**,**H**), Fundus and wide-angle fundus photographs of eye 4 taken 7 weeks before and after symptom exacerbation. The patient experienced reduced visual acuity 6 days after the first dose of BNT162b2; a massive submacular hemorrhage was evident.

Table 2 lists the characteristics of patients with AMD-related submacular hemorrhage. Figure 1 and Figure 2 present representative cases. Twelve eyes of 11 patients (five men and six women) were included. The median age at symptom presentation was 81 years (range: 62–84 years). Six patients (55%) had diabetes mellitus and another six had hypertension. Eight patients (73%) had received the Pfizer vaccine (BNT162b2) and two (18%) the AstraZeneca (ChAdOx1) vaccine. Eight patients (73%) experienced ocular disease exacerbation after their first vaccination and two (18%) experienced disease exacerbation after their second vaccination. The median time between vaccination and symptom exacerbation was 2.0 days (interquartile range: 1.0–3.0 days). Three eyes (25%) were observed and five received intravitreal bevacizumab injections (42%). C3F8 gas injection was performed in two eyes (17%), bevacizumab and C3F8 gas were injected into one eye (8%), and ranibizumab was intravitreally injected into one eye (8%). The median logMAR before symptom development was 0.82 (interquartile range: 0.52–1.40) and that at symptom presentation was 1.40 (interquartile range: 0.77–1.62).

### 3.2. Retinal Vein Occlusion after SARS-CoV-2 Vaccination

**Figure 3:**
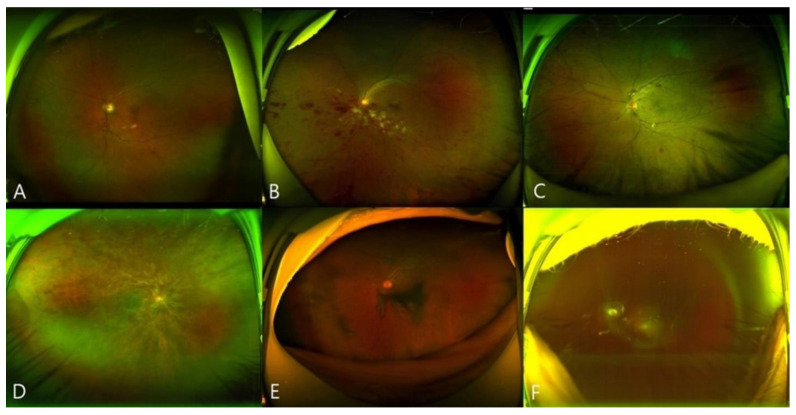
Images of representative eyes exhibiting retinal vein occlusion (RVO) after vaccination. (**A**,**B**), Wide-field fundus photographs revealing branch RVO (eyes 14 and 21). (**C**,**D**), Wide-field fundus photographs revealing central RVO (eyes 23 and 17). (**E**,**F**), Wide-field fundus photographs of RVO with vitreous hemorrhage (eyes 22 and 13).

Table 3 lists the characteristics of the patients with RVO. Figure 1 and Figure 3 present representative cases. Eleven eyes of 11 patients (six men and five women) were included. The median age at symptom presentation was 68 years (range: 51–85 years). Three patients (27%) had diabetes mellitus and eight (73%) had hypertension. Six patients (55%) received the AstraZeneca (ChAdOx1) vaccine and five (46%) the Pfizer vaccine (BNT162b2). Nine patients (82%) experienced ocular disease exacerbation after their first vaccination and two (18%) after their second vaccination. The median time between vaccination and symptom exacerbation was 2.0 days (interquartile range: 1.0–3.0 days). Five eyes (46%) were observed, and bevacizumab was intravitreally injected into six eyes (55%). The mean logMAR before symptom development in five eyes was 0.10 (interquartile range: 0.00–0.70) and that at symptom presentation was 1.05 (interquartile range 0.10–2.00). Three (27%) eyes exhibited both vitreous and retinal hemorrhage. Compared to the submacular hemorrhage patients, the mean age of the RVO patients was significantly lower (*p* = 0.027, Mann–Whitney U test). The logMAR prior to exacerbation in RVO patients was significantly lower than the logMAR of the submacular hemorrhage patients (*p* = 0.027), although many patients with submacular hemorrhage had previously been diagnosed with AMD. The logMAR after symptom presentation did not differ significantly between the two groups (*p* = 0.413). The time from vaccination to symptom exacerbation did not differ significantly between the submacular hemorrhage and RVO patients (*p* = 0.512).

## 4. Discussion

To the best of our knowledge, this is the largest case series examining RVO and AMD-related submacular hemorrhage after SARS-CoV-2 vaccination. Known ocular complications associated with vaccination include acute macular neuroretinopathy after ChADOx1 nCOV-19 vaccination [14] and central serous retinopathy, reactivation of Vogt–Koyanagi–Harada disease, panuveitis, and DMEK graft rejection after BNT162b2 vaccination [15,16,17,18]. No report has described RVO or AMD-related submacular hemorrhage, nor any other ocular hemorrhagic complications, after vaccination. 

A few reports of retinal hemorrhage after other vaccinations have appeared. Two case reports on RVO developing after hepatitis B vaccination with the hepatitis B surface antigen gene were published in the 1990s, [23,24] but there have been none since then. Binenbaum et al. [25] studied 5177 vaccinated children aged from 1 to 23 months, and found that 9 developed retinal hemorrhage, but all of these had suffered abusive head trauma. No temporal association between vaccination and retinal hemorrhage developing within the next 21 days was evident. There have been a few case reports on central retinal vein occlusion (CRVO) after SARS-CoV-2 vaccination. A 52-year-old male was diagnosed with CRVO 15 days after receiving the first dose of BNT162b2 vaccination [21]. A 50-year-old healthy patient felt immediate visual reduction in his left eye during the 15 min of mandatory surveillance after receiving his second dose of BNT162b2, and was revealed to have CRVO 20 days after the date of vaccination [22]. A 54-year-old female was referred to an ophthalmology clinic 8 days after her second dose of mRNA-1273, and was diagnosed with combined central retinal artery occlusion and CRVO. She felt the symptoms 2 days after the day of vaccination, and her vision was that of no light perception at presentation [20].

Based on our data, there seems to be a temporal link between SARS-CoV-2 vaccination and RVO and AMD-related submacular hemorrhage. The median time from vaccination to symptom onset was 2 days, and all patients for whom the time of exacerbation was noted developed hemorrhage within 4 weeks of vaccination. Many of our patients were referrals from other centers (our institute is one of the largest tertiary centers in Korea), but the high number of patients with RVO and AMD-related submacular hemorrhage encountered within a short period (~4 months) indicates that these uncommon pathologies did not develop by chance. Submacular hemorrhage is known to occur in 5.4 per million, which indicates that this is not a condition seen every day in a retina clinic [26]. According to a study including 791 newly diagnosed neovascular AMD patients, 129 eyes (16.3%) exhibited submacular hemorrhage at initial presentation, and the incidence of submacular hemorrhage was greater in PCV (23.6%, 78 of 330 eyes) than in typical neovascular AMD (9.4%, 28 of 297 eyes) [27]. The fact that only one eye had a history of previous PCV in the submacular hemorrhage after vaccination group suggests the possibility of different clinical characteristics of such conditions. Furthermore, as of July 31 2021, when our last patient was enrolled, only 33.2% of the South Korean population had been vaccinated at least once and only 13.5% had been fully vaccinated. Therefore, 21 cases in less than 4 months from among a limited number of vaccinated persons cannot be considered coincidental, especially given that this was a single-center study. 

VITT and CVST developing after ChAdOx1 nCoV-19 vaccination have been confirmed in several reports [8,9]; it was important to determine if our cases were associated with VITT, which is diagnosed when serum anti-PF4 Ab is detected by ELISA [8]. Five of our patients (two vaccinated with BNT162b2 nCoV-19 and three with ChAdOx1 nCoV-19) underwent anti-PF4 Ab tests; all were negative. Thus, an association between VITT and retinal/submacular hemorrhage in our cohort seems unlikely. However, it may be that the anti-PF4 Ab titer was too low for clinical detection, although sufficiently high for thromboses to form in retinal vessels narrower than those of most other organs. 

Pegylated interferon-associated retinopathy is a well-known condition [28,29,30]. Pegylation of polypeptides such as interferon with polyethylene glycol (PEG) improves pharmacodynamic and pharmacokinetic profiles, but PEG has been associated with RVO, retinal artery occlusion, ischemic ophthalmopathy, and Vogt–Koyanagi–Harada disease, especially in patients with underlying diabetes or hypertension (who are susceptible to the development of microvascular abnormalities). BNT162b2 is an mRNA vaccine that contains PEG; it is thus possible that the RVO and AMD-related submacular hemorrhages seen in our patients were caused by the PEG in the BNT162b2 vaccine, similar to pegylated interferon. The fact that over 80% of our patients had diabetes or hypertension (17 patients, 81%) lends credibility to this hypothesis. However, PEG-interferon-associated retinopathy usually develops bilaterally, without symptoms, 2–6 months after treatment commences, whereas our patients were predominantly unilateral and symptomatic, and developed disease within 21 days of vaccination. Furthermore, unlike the BNT162b2 vaccine, ChAdOx1 nCoV-19 is not an mRNA vaccine; rather, it is an adenovirus-vectored vaccine that includes polysorbate 80 as an excipient; this material appears to be safe even when directly injected into the vitreous cavity [31]. RVO and submacular hemorrhage also developed after ChAdOx1 vaccination. 

As more than 80% of our patients (17 of 21) had diabetes or hypertension, our findings suggest that eyes more vulnerable to microvascular dysfunction are also more likely to develop hemorrhagic complications after SARS-CoV-2 vaccination. Thus, the complications could be regarded as reactions in “at-risk” patients. Both the adenoviral and mRNA-based vaccines generate spike proteins; the trigger could be the spike proteins themselves or the immune reaction elicited by their presence. Molecular mimicry between the spike protein and a self-antigen could stimulate platelet activation and retinal hemorrhage. 

Most systemic drug-related retinal toxicity is bilateral, but the RVO and AMD-related submacular hemorrhage in our patients were mostly unilateral. Submacular hemorrhage developed in eyes with pre-existing wet AMD, and it may be that hemorrhage developed in at-risk eyes with vulnerable vessels. Recently, there has been a multi-center-based report on cases of RVO and retinal hemorrhage in COVID-19 patients in Spain [32]. As 36 out of 39 RVO cases in COVID-19 patients occurred unilaterally, it seems possible that host response to the spike protein of SARS-CoV-2 virus is affecting at-risk eyes, leading to retinal vascular abnormality. RVO typically develops unilaterally, even in patients with diabetes and hypertension; the absence of bilateral RVO does not rule out a vaccine-induced condition. The pathogenesis of retinal hemorrhages developing after SARS-CoV-2 vaccination requires further study. 

All patients had received either the adenoviral vector vaccine ChAdOx1 (AstraZeneca) or the mRNA vaccine BNT162b2 (Pfizer-BioNTech). This may be because these two vaccines were the mainstays of the government-driven, large-scale vaccination program initiated in South Korea. Both vaccines were associated with RVO and submacular hemorrhage. As the vaccines were given to different populations (in accordance with government guidelines), direct comparison between them might be associated with selection bias. For ChAdOx1, the age limit was changed from over 30 years to over 50 years during the study period. No restriction was placed on BNT162b2, apart from the manufacturer’s guideline, i.e., that vaccine recipients should be aged over 16 years.

The limitations of our study included the retrospective design, small number of cases, and lack of complete blood screening data for all patients. As the study lacked an adequate control group, only observational conclusions can be made. Nevertheless, to the best of our knowledge, this is the largest case series on RVO and AMD-related submacular hemorrhage developing after vaccination against SARS-CoV-2. Further studies with larger patient groups for evaluation of direct mechanisms and the extent of the effect of SARS-CoV-2 vaccination on retinal hemorrhage are necessary. Nevertheless, clinicians should be aware of the possibility of such cases, and it may be necessary to adjust the treatment schedules of high-risk RVO and AMD patients.

## Figures and Tables

**Table 1:** Demographics of patients who developed submacular hemorrhage and/or retinal vein occlusion after COVID-19 vaccination.

Category	Value
**Sex**	
Male	11 (52%)
Female	10 (48%)
**Age (Median)**	77 (range: 51–85 years)
**Laterality**	
Unilateral	19 (90%)
Bilateral	2 (10%)
**Diagnosis (Eyes)** AMD	12 (52%)
RVO	11 (48%)
**Medical History**	
DM	9 (43%)
HTN	11 (48%)
**Type of Vaccination**	
Pfizer (BNT162b2)	12 (61%)
AstraZeneca (ChAdOx1)	8 (38%)
**Number of Vaccination**	
1st	17 (81%)
2nd	4 (19%)
**Visual Acuity before Symptom Development (logMAR)**	0.76 (interquartile range: 0.27–1.23)
**Visual Acuity at Symptom Presentation (logmar)**	1.40 (interquartile range 0.52–1.70)
**Time between Vaccination and Symptom Aggravation**	2.0 days (interquartile range: 1.0–3.0 days).

AMD = age-related macular degeneration; DM = diabetes mellitus; HTN = hypertension; LogMAR = logarithm of the minimal angle of resolution.

**Table 2:** Summary of the characteristics of patients who developed submacular hemorrhage after COVID-19 vaccination.

Eye Number	Age, Years	Gender	Laterality	Underlying Disease	Underlying Ocular History	Type of Vaccination	Number of Vaccination	Interval of Vaccine Inoculation to Symptom Development (Days)	Treatment	Hematologic Abnormality	Anti-PF4 Antibody	Visual Acuity before Symptom Development *	Visual Acuity at Symptom Development *
1	82	F	OD	HTN DM Hypothyroidism Syphillis	AMD 53 Anti-VEGF injections (Last 7 days before)	Pfizer	1	3	Observation	Not tested	Not tested	0.2	0.02
2	82	F	OS	HTN DM Hypothyroidism Syphillis	AMD 53 Anti-VEGF injections (Last 25 months before)	Pfizer	1	3	Observation	Not tested	Not tested	0.04	0.04
3	81	M	OD	HTN Atrial fibrillationCAOD Spinal stenosis HCMP	Cataract surgery	Pfizer	1	10	C3F8	Not tested	Not tested	0.4	FC10
4	81	M	OS	None	Cataract surgery	Pfizer	2	6	C3F8	Not tested	Not tested	0.02	FC10
5	82	F	OD	DM HTN Angina	Cataract surgeryVitrectomy for vitreous hemorrhage	Pfizer	1	3	Anti-VEGF (Bevacizumab), C3F8	Not tested	Not tested	0.15	0.04
6	84	F	OS	Polycystic kidney	AMD23 Anti-VEGF injections (Last 5 months before)	Pfizer	2	15	Anti-VEGF (Bevacizumab)	Not tested	Not tested	0.1	0.1
7	77	F	OD	HTN HBV carrier Sigmoid colon cancer on chemotherapy s/p low anterior resection	Cataract surgery	Pfizer	1	16	Anti-VEGF (Ranibizumab)	Not tested	Not tested	-	0.2
8	70	M	OD	DM	AMD 13 Anti-VEGF injections (Last 4 months before)	NA	1	Within 4 weeks ^†^	Anti-VEGF (Bevacizumab)	Not tested	Not tested	0.15	0.04
9	82	M	OS	DMHTN	PCV Cataract surgery26 Anti-VEGF injections (Last 7 months before) Photodynamic therapy	Pfizer	1	14	Anti-VEGF (Bevacizumab)	Not tested	Not tested	0.05	0.04
10	71	F	OD	Dyslipidemia	AMD 2 Anti-VEGF injections (Last 6 months before)	AstraZeneca	1	2	Anti-VEGF (Bevacizumab)	Not tested	Not tested	0.6	0.3
11	80	F	OD	DM HTN	AMD 12 Anti-VEGF injections (Last 15 months before)	Pfizer	1	Within 4 weeks ^†^	Anti-VEGF (Bevacizumab)	Not tested	Not tested	0.04	0.04
12	62	M	OD	DM	none	AstraZeneca	1	Within 4 weeks ^†^	Observation	Not tested	Not tested	0.3	0.3

AMD = age-related macular degeneration; CAOD = coronary arterial occlusive disease; DM = diabetes mellitus; F = female; HBV = hepatitis virus B; HCMP = hypertrophic cardiomyopathy; HTN = hypertension; M = male; NA = not available; OD = oculus dexter; OS = oculus sinister; PCV = polypoidal choroidal vasculopathy; VEGF = vascular endothelial growth factor; * Visual acuity was demonstrated with decimal system. ^†^ Patients were unable to report the exact time point of symptom development.

**Table 3:** Summary of the characteristics of patients who developed retinal vein occlusion after COVID-19 vaccination.

Eye Number	Age, Years	Gender	Laterality	Underlying Disease	Underlying Ocular History	Type of Vaccination	Number of Vaccination	Interval of Vaccine Inoculation to Symptom Development (Days)	Treatment	Hematologic Abnormality	Anti-PF4 Antibody	Visual Acuity before Symptom development *	Visual Acuity at Symptom Development *
13	68	F	OS	Dyslipidemia	Vitrectomy for vitreous hemorrhage Cataract surgery	AstraZeneca	1	1	Observation	Not tested	Not tested	-	HM
14	76	M	OS	HTN	NTG Cataract surgery	Pfizer	1	3	Observation	Tested with negative result	Tested with negative result	1	0.8
15	85	F	OD	DM HTN ESRD Old tuberculosis Dementia	Vitrectomy with cataract surgery for vitreous hemorrhage	Pfizer	2	1	Anti-VEGF (Bevacizumab)	Not tested	Not tested	0.1	FC10
16	59	M	OS	DM HTN	Vitrectomy with secondary IOL scleral fixation	AstraZeneca	1	2	Observation	Tested with negative result	Tested with negative result	0.8	0.8
17	61	M	OD	None	None	AstraZeneca	1	2	Anti-VEGF (Bevacizumab)	Tested with negative result	Tested with negative result	-	0.04
18	79	M	OS	DM Early gastric cancer	None	Pfizer	2	2	Anti-VEGF (Bevacizumab)	Tested with negative result	Tested with negative result	-	0.04
19	77	F	OS	HTN HBV carrier Sigmoid colon cancer on chemotherapy s/p low anterior resection	Cataract surgery	Pfizer	1	16	Anti-VEGF (Bevacizumab)	Not tested	Not tested	-	0.8
20	63	M	OD	DM	DME Intravitreous triamcinolone injection	Pfizer	1	13	Anti-VEGF (Bevacizumab)	Tested with low Hb, Hct	Tested with negative result	0.4	0.01
21	51	F	OS	HTN	None	AstraZeneca	1	21	Anti-VEGF (Bevacizumab)	Not tested	Not tested	-	0.09
22	81	F	OS	HTN	Cataract surgery	Pfizer	1	4	Observation	Not tested	Not tested	-	0.3
23	61	M	OS	HTN	Uveitis	AstraZeneca	1	3	Observation	Not tested	Not tested	1	0.9

AMD = age-related macular degeneration; DM = diabetes mellitus; DME = diabetic macular edema; ESRD = end-stage renal disease; F = female; HBV = hepatitis virus B; HTN = hypertension; IOL = intraocular lens; M = male; NTG = normal tension glaucoma; OD = oculus dexter; OS = oculus sinister; VEGF = vascular endothelial growth factor; * Visual acuity was demonstrated with decimal system.

## Data Availability

No new data were created or analyzed in this study. Data sharing is not applicable to this article.
